# Hepatitis C Virus Strain-Dependent Usage of Apolipoprotein E Modulates Assembly Efficiency and Specific Infectivity of Secreted Virions

**DOI:** 10.1128/JVI.00422-17

**Published:** 2017-08-24

**Authors:** Romy Weller, Kathrin Hueging, Richard J. P. Brown, Daniel Todt, Sebastian Joecks, Florian W. R. Vondran, Thomas Pietschmann

**Affiliations:** a Institute of Experimental Virology, Twincore, Centre for Experimental and Clinical Infection Research, Hanover, Germany; b Department of General, Visceral and Transplant Surgery, Hanover Medical School, Hanover, Germany; c German Centre for Infection Research, Partner Site Hanover-Braunschweig, Hanover, Germany; Washington University School of Medicine

**Keywords:** ApoE, apolipoprotein, assembly, genotypes, hepatitis C virus

## Abstract

Hepatitis C virus (HCV) is extraordinarily diverse and uses entry factors in a strain-specific manner. Virus particles associate with lipoproteins, and apolipoprotein E (ApoE) is critical for HCV assembly and infectivity. However, whether ApoE dependency is common to all HCV genotypes remains unknown. Therefore, we compared the roles of ApoE utilizing 10 virus strains from genotypes 1 through 7. ApoA and ApoC also support HCV assembly, so they may contribute to virus production in a strain-dependent fashion. Transcriptome sequencing (RNA-seq) revealed abundant coexpression of ApoE, ApoB, ApoA1, ApoA2, ApoC1, ApoC2, and ApoC3 in primary hepatocytes and in Huh-7.5 cells. Virus production was examined in Huh-7.5 cells with and without ApoE expression and in 293T cells where individual apolipoproteins (ApoE1, -E2, -E3, -A1, -A2, -C1, and -C3) were provided in *trans*. All strains were strictly ApoE dependent. However, ApoE involvement in virus production was strain and cell type specific, because some HCV strains poorly produced infectious virus in ApoE-expressing 293T cells and because ApoE knockout differentially affected virus production of HCV strains in Huh-7.5 cells. ApoE allelic isoforms (ApoE2, -E3, and -E4) complemented virus production of HCV strains to comparable degrees. All tested strains assembled infectious progeny with ApoE in preference to other exchangeable apolipoproteins (ApoA1, -A2, -C1, and -C3). The specific infectivity of HCV particles was similar for 293T- and Huh-7.5-derived particles for most strains; however, it differed by more than 100-fold in some viruses. Collectively, this study reveals strain-dependent and host cell-dependent use of ApoE during HCV assembly. These differences relate to the efficacy of virus production and also to the properties of released virus particles and therefore govern viral fitness at the level of assembly and cell entry.

**IMPORTANCE** Chronic HCV infections are a major cause of liver disease. HCV is highly variable, and strain-specific determinants modulate the response to antiviral therapy, the natural course of infection, and cell entry factor usage. Here we explored whether host factor dependency of HCV in particle assembly is modulated by strain-dependent viral properties. We showed that all examined HCV strains, which represent all seven known genotypes, rely on ApoE expression for assembly of infectious progeny. However, the degree of ApoE dependence is modulated in a strain-specific and cell type-dependent manner. This indicates that HCV strains differ in their assembly properties and host factor usage during assembly of infectious progeny. Importantly, these differences relate not only to the efficiency of virus production and release but also to the infectiousness of virus particles. Thus, strain-dependent features of HCV modulate ApoE usage, with implications for virus fitness at the level of assembly and cell entry.

## INTRODUCTION

Hepatitis C virus (HCV) is a hepatotropic, plus-strand RNA virus which is extraordinarily variable. According to phylogenetic analyses, HCV isolates are classified into seven distinct genotypes which differ from each other by more than 30% at the nucleotide level. Moreover, HCV genotypes are further subdivided into 67 confirmed and 20 provisional subtypes (1, 2). HCV is transmitted mainly parenterally through the transcutaneous route. Upon contact with the virus, the majority of exposed individuals progress to a chronically persistent infection, which over the course of decades can lead to severe liver disease, including hepatitis, cirrhosis, and hepatocellular carcinoma (3, 4). HCV genotypes and subtypes have a distinct geographical distribution. For example, HCV infections in industrialized countries are dominated by subtype 1a, 1b, 2a, and 3a viruses, which were spread by contaminated blood products prior to HCV's discovery and which now are transmitted primarily among men who have sex with men and among people who inject drugs (5–7). The remaining genotypes, whose historical spread is less well characterized, are frequently observed in West Africa (primarily genotypes 1 and 2), Central Africa and the Middle East (genotype 4), southern Africa (genotype 5), South Asia (genotype 3), and Southeast Asia (genotype 6) (5, 8, 9). HCV genotypes are associated with distinct disease progression and response to therapy. For example, HCV genotype 3 infection is associated with accelerated fibrosis progression and a higher risk of developing cirrhosis, steatosis, and hepatocellular carcinoma, while genotype 2 is associated with less severe fibrosis (10–12). However, the molecular mechanisms responsible for genotype-dependent differences in the natural course of HCV infection are poorly understood.

The recent licensing of direct-acting antivirals (DAAs) has revolutionized HCV patient care (13, 14). However, viral reinfection is possible, and the high costs of these drugs limit access to therapy, particularly in resource-poor countries. Thus, global control of the HCV disease burden would benefit from the development of a prophylactic vaccine. However, the pronounced variability of HCV poses a challenge for development of vaccination strategies eliciting protective immunity across all major HCV genotypes (15). Successful vaccine development can therefore be facilitated by a broader understanding of virus-host interactions, which can potentially differ between HCV genotypes.

HCV carries a single open reading frame (ORF) encoding a length of about 3,000 amino acids (16). Individual viral proteins are liberated from the polyprotein by cellular proteases and two viral peptidases. Multiple steps of the HCV replication cycle are closely linked with host cell lipid metabolism and membrane-remodeling processes (17). Moreover, HCV particles are rich in lipids akin to those present in very-low-density lipoproteins (VLDLs), they have a low buoyant density, and they circulate in close association with human lipoproteins, including lipoprotein components such as apolipoprotein A (ApoA), ApoB, ApoC, and ApoE (18–24). Due to these features, HCV particles have been named “lipoviro particles” (LVPs) (24) The association of HCV with ApoE has been reported to facilitate HCV cell entry through augmented binding of virus particles to heparin sulfate proteoglycans, which facilitates virus attachment to the cell surface (25, 26). Furthermore, this interplay was shown to contribute to immune evasion by protecting glycoprotein target sites from neutralizing antibodies (25, 27–29).

Several independent studies have highlighted the importance of human ApoE for assembly and release of infectious HCV progeny from infected cells (28, 30–32). Human non-liver cell lines (e.g., HEK-293T) that lack expression of ApoE are refractory to HCV production unless ApoE is provided ectopically, thus emphasizing the critical role of ApoE during HCV particle production (33, 34). It has been shown that ApoE likely facilitates a late assembly step after membrane envelopment of HCV capsids and that ApoE is also critical for viral cell-to-cell transmission (33, 35, 36). While it is not fully understood how ApoE mediates HCV assembly, direct interactions of ApoE with the glycoproteins E1 and E2 and the nonstructural protein 5A have been described (31, 33, 34, 37–39). Intriguingly, alternative structurally related human apolipoproteins, including various members of the ApoC and ApoA family, and even a single non-lipoprotein-derived amphipathic alpha helix connected with a signal peptide, compensate for the lack of ApoE expression and partly rescue infectious HCV production in the absence of ApoE (40–42). These data suggest that structural and functional features of endoplasmic reticulum (ER)-targeted amphipathic alpha helices are important for HCV particle production, possibly in the absence of a direct interaction with viral proteins.

To date, studies dissecting the role of apolipoproteins in the HCV replication cycle have utilized a restricted panel of isolates which underrepresents the global diversity of HCV. Therefore, we explored the ApoE dependence of representative HCV isolates from all major genotypes to identify potential isolate-specific differences in HCV assembly and secretion pathways.

## RESULTS

### Comparative analysis of virus production reveals strain-dependent differences in utilization of human ApoE among HCV chimeras.

**FIG 1:**
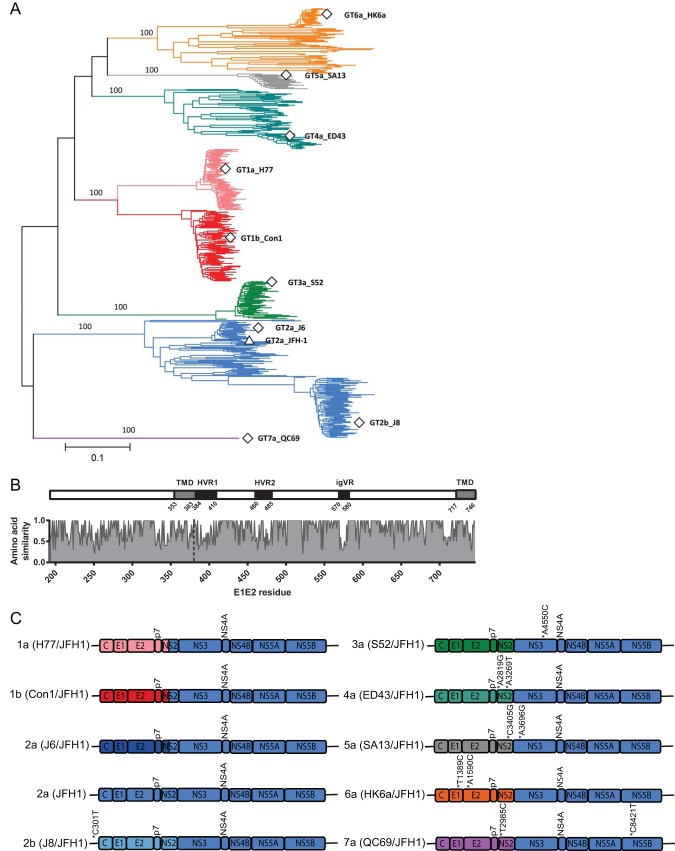
HCV genetic and amino acid diversity. (A) E1E2 phylogenetic tree depicting the evolutionary relationships of the seven HCV genotypes, with genotypes color coded (subtypes 1a/1b, pink/red; genotype 2. blue; genotype 3, green; genotype 4, turquoise; genotype 5, gray; genotype 6, orange; genotype 7, purple). The positions of E1E2s derived from the nine chimeric strains utilized in this study are highlighted with open squares. The position of the JFH-1 E1E2 is marked with an open triangle. Branch lengths represent genetic distance measured in nucleotide substitutions per site and are proportional to the scale bar. Bootstrap values are assigned to the branches leading to the seven genotypes and are percentages derived from 1,000 replications. (B) Amino acid similarity plot of full-length HCV envelope glycoproteins derived from the 10 strains utilized in this study, with relative similarity shown on the *y* axis and amino acid position in the encoded proteins presented on the *x* axis. For the purpose of positional referencing, a cartoon of the E1E2 protein is located directly above, with the three hypervariable regions of E2 (HVR1, HVR2, and igVR) highlighted in black and the E1 and E2 transmembrane domains (TMD) highlighted in gray. The dashed vertical line represents the E1/E2 boundary. All numbering is relative to the full-length ORF position in the H77 reference strain (accession number NC_004102). (C) HCV constructs used in this study. The colors of genome portions matches the colors chosen for display of distinct HCV genotypes and subtypes in panel A. Asterisks indicate adaptive mutations.

To determine whether the function of human ApoE as a crucial host factor for assembly and release of infectious HCV particles is conserved across representative isolates of all major HCV genotypes, we took advantage of HCV JFH1 and nine JFH1-based chimeras that represent the diversity of globally sampled HCV (Fig. 1) (43–47). The sequences utilized in this study were incorporated into a phylogenetic analysis with globally sampled isolates representing the major subtypes and genotypes (Fig. 1A). The 10 strains utilized are distributed throughout the phylogeny and are therefore representative of the breadth of genetic diversity apparent worldwide. This diversity extends to the amino acid level, as demonstrated by comparison of translated E1E2 proteins for the 10 strains (Fig. 1B). While extreme diversity is apparent in the 3 hypervariable regions of E2, both functionally conserved domains and additional variable regions are distributed throughout the E1E2-coding region (Fig. 1B). An overview of the constructs used, including adaptive mutations and intra- or intergenotypic fusion sites, is schematically depicted in Fig. 1C.

**FIG 2:**
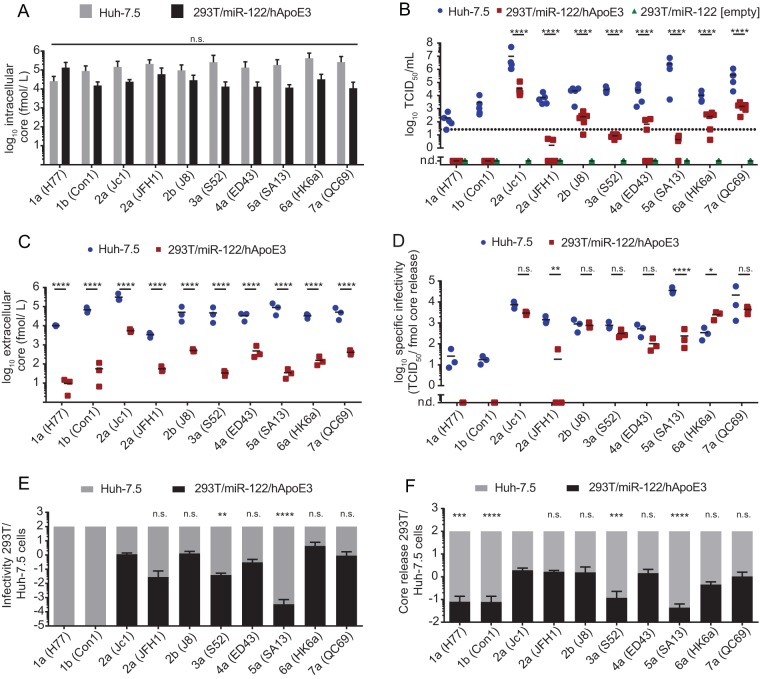
Strain-dependent usage of ApoE3 during HCV assembly in 293T/miR-122 cells. (A) Huh-7.5 cells and non-liver-derived 293T/miR-122 cells expressing hApoE3 were transfected with *in vitro*-transcribed RNA of the depicted HCV constructs, and intracellular core protein was quantified 48 h later to compare transfection, translation, and replication efficacy by use of a core-specific ELISA. Depicted are means and standard deviations from three independent experiments (n.s., not significant by 2-way ANOVA followed by Sidak's multiple-comparison test). (B) Infectious virus production from these transfected cells was quantified by titrating the cell-free culture fluids collected at 48 h posttransfection and by using endpoint dilution assay on Huh-7.5 target cells. Infectivity is given as 50% tissue culture infectious dose per milliliter (TCID_50_/ml). 293T/miR-122 cells lacking ApoE expression were transfected with each HCV chimera in parallel, and no infectious events were detected. The dotted line represents the lower limit of quantification (LLOQ) of the assay. Displayed are individual results of four to six independent experiments, with the mean presented as a horizontal bar. Mean TCID_50_s in Huh-7.5 cells were compared to infectivity in 293T/miR-1227hApoE3 cells for each strain (****, *P* < 0.0001; n.d., not detected [by 2-way ANOVA followed by Sidak's multiple-comparison test]). (C) At 48 h after transfection, secretion of core protein into the cell culture supernatant as an indicator of particle release was additionally quantified by core-specific ELISA. Results from three independent experiments, with the mean presented as a horizontal bar, are given. Mean concentrations of core in Huh-7.5 were compared to detected particles in 293T/miR-122/hApoE3 cells for each strain (****, *P* < 0.0001 by 2-way ANOVA followed by Sidak's multiple-comparison test). (D) Based on the data plotted in panels B and C, the specific infectivity (i.e., the TCID_50_ units per fmol of released core protein) was calculated in three independent experiments. Mean specific infectivities in Huh-7.5 cells were compared to those in 293T/miR-122/hApoE3 cells for each strain (****, *P* < 0.0001; **, *P* < 0.01; *, *P* < 0.05; n.s., not significant; n.d., not detected [by 2-way ANOVA followed by Sidak's multiple-comparison test]). (E and F) Efficiencies of infectious virus particle production (E) and core protein release (F) from 293T/miR-122/hApoE3 cells were calculated and displayed, with those observed in Huh-7.5 cells normalized to 100%. Significant differences of relative infectivity and relative core release in 293T/miR-122/hApoE3 cells of different chimeras compared to Jc1 are indicated (****, *P* < 0.0001; ***, *P* < 0.001; **, *P* < 0.01; n.s., not significant [by 1-way ANOVA followed by Sidak's multiple-comparison test]).

Since primary human hepatocytes (PPHs) (41) and the human hepatoma cell line Huh-7.5 express abundant mRNA levels of various exchangeable apolipoproteins (see Fig. 4A), we first examined HCV infectious particle production in non-liver-derived 293T/miR-122 cells ectopically expressing ApoE3 (33, 41) to specifically assess the role of ApoE in virus production. As a reference, highly permissive Huh-7.5 cells were transfected in parallel. Virus RNA translation and replication were determined by quantification of intracellular HCV core protein expression using a commercial enzyme-linked immunosorbent assay (ELISA) 48 h after transfection (Fig. 2A), and infectious virus production was measured by using a limiting-dilution assay (Fig. 2B). 293T/miR-122 cells expressing an empty vector served as a negative control. Furthermore, release of particles was quantified by assessment of extracellular core protein quantities at this time point (Fig. 2C). Similar intracellular amounts of core protein were detected for all HCV constructs in transfected 293T/miR-122/hApoE3 cells, indicating comparable transfection, RNA genome translation, and replication efficiencies. The abundance of HCV core was also comparable for HCV-transfected Huh-7.5 cells, and it was ca. 2- to 10-fold higher in Huh-7.5 cells than in 293T/miR122/hApoE3 cells, suggesting higher HCV transfection and/or replication efficiency in the former cells (Fig. 2A). Huh-7.5 cell-derived virus titers varied between the different chimeras, with genotypes 2a (Jc1) and 5a (SA13) yielding the highest infectivity (1.1 × 10^7^ 50% tissue culture infective doses [TCID_50_]/ml and ∼1.1 × 10^6^ TCID_50_/ml, respectively) and the 1a (H77) and 1b (Con1) chimeras reaching the lowest infectivity (8.2 × 10^1^ TCID_50_/ml and 2.9 × 10^3^ TCID_50_/ml, respectively) (Fig. 2A). This was expected and roughly reflects the fitness of these chimeras as reported in previous studies (43–47). All chimeras yielded significantly less infectious virus upon transfection of 293T/miR-122/hApoE3 cells than upon transfection of Huh-7.5 cells. Nevertheless, relative to infectious virus production in Huh-7.5 cells, some HCV chimeras produced much less infectivity in 293T/miR-122/hApoE3 cells than expected. For instance, genotype 5a (SA13) grew to higher titers upon transfection of Huh-7.5 cells, but virus production was below the lower limit of quantification (LLOQ) upon transfection of 293T/miR-122/hApoE3 cells and was thus reduced by at least 500,000-fold (Fig. 2B and E). In contrast, genotype 2a (Jc1) also yielded relatively high virus titers upon transfection of 293T/miR-122/hApoE3 cells, which were only ca. 300-fold lower than the ones reached upon transfection of Huh-7.5 cells. Thus, these results suggest strain-specific differences in utilizing ApoE from non-liver cells. This may be due to direct or indirect effects caused by other host factors expressed (or not expressed) in 293T/miR122/hApoE3 cells.

HCV core protein release was detectable in each case and, as expected, was significantly lower for each chimera after transfection of 293T/miR122/hApoE3 cells than after transfection of Huh-7.5 cells (Fig. 2C). When comparing the relative efficiency of core protein release of each of these chimeras between these different cell lines, we noted major differences (Fig. 2F). In case of Jc1, the difference in core release between these cell lines was roughly 50-fold, which matches the difference in infectivity. In contrast, for the genotype 5a chimera (SA13), the difference in core release was more than 2,000-fold. This observation suggested that HCV chimeras differ in their capacity to release HCV core protein in these two cell lines. As mentioned above, genotype 5a (SA13) produced more than 500,000-fold fewer infectious particles upon transfection of 293T/miR-122/ApoE3 cells, but core release was attenuated only ca. 2,000-fold. Thus, for this chimera also, the specific infectivity (that is, the level of infectiousness associated with a given quantity of released core protein) was much lower upon transfection of 293T/miR122/hApoE3 cells than upon transfection of Huh-7.5 cells (Fig. 2D). Note that for the genotype 5a (SA13) chimera, we can only estimate the maximal specific infectivity of 293T/miR-122/hApoE3-derived viruses because the infectivity measurements were below the lower limit of quantification. Therefore, the true specific infectivity of these particles may be even lower. For JFH1 also, the specific infectivity of 293T/miR122/hApoE3-derived particles was lower than that of particles from Huh-7.5 cells. In contrast, many other chimeras, for instance, genotype 2a Jc1, 2b (J8), 3a (S52), or 7a (QC69), produced particles with comparable specific infectivity upon transfection of these cell lines, and uniquely the specific infectivity of 6a (HK6a) particles was significantly higher after transfection of 293T/miR-122/hApoE3 cells than after transfection of Huh-7.5 cells (Fig. 2D). Taken together, this analysis confirms previous reports indicating different assembly competencies of these chimeras in human liver cell lines (43–47). Unexpectedly, these differences in fitness are not precisely mirrored in 293T/miR-122/hApoE3 cells, as some chimeras are much more attenuated in assembly and release of viral progeny than others (e.g., genotype 5a [SA13] compared to 2a [Jc1]) and because the specific infectivity of released particles also varied. Collectively, these results suggested that HCV chimeras differ in host factor requirements, possibly in their ApoE usage, for infectious particle assembly and release.

### Comparable utilization of ApoE isoforms in assembly and virus production by HCV chimeras.

**FIG 3:**
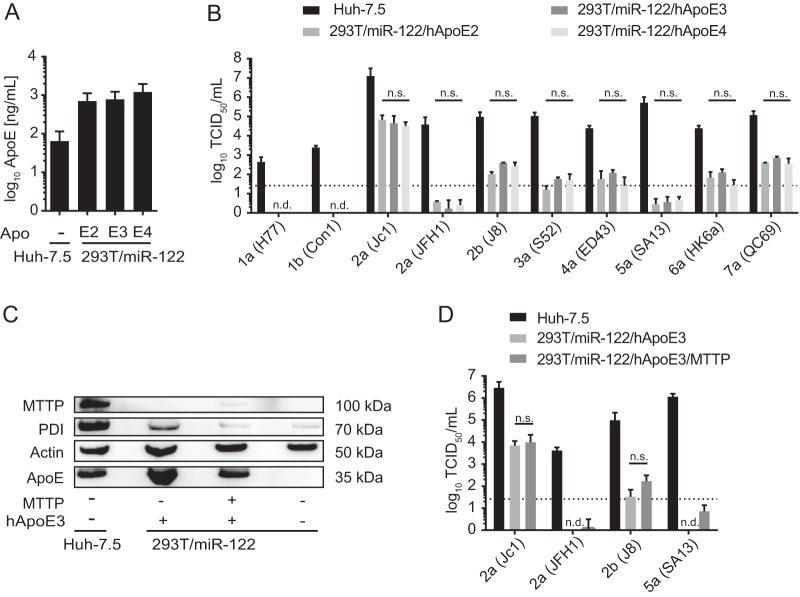
Ability of ApoE allelic isoforms and MTTP to support infectious virus production of HCV chimeras in 293T/miR-122 cells. (A) 293T/miR-122 cells were transduced to express the allelic isoforms of ApoE, and secretion of these allelic forms (ApoE2, ApoE3, and ApoE4) was quantified by using an ApoE ELISA. (B) These cell lines were subsequently transfected with RNAs of the indicated HCV constructs, and release of infectious particles was determined by a limiting dilution assay. Differences in infectivity observed among the three cell lines were analyzed by 2-way ANOVA followed by Sidak's multiple-comparison test (n.s., not significant; n.d., not detected). (C) 293T/miR-122/hApoE3 cells were lentivirus transduced to express the additional host factor MTTP. Protein expression of MTTP as well as the protein disulfide isomerase (PDI), which completes the heterodimeric MTTP, was confirmed by immunoblotting using antibodies specific for MTTP, PDI, β-actin, and ApoE. (D) Cells were transfected with the indicated HCV constructs. Infectious virus production was assessed by a limiting dilution assay. Differences in means were analyzed by multiple *t* tests, corrected with the Holm-Sidak method (n.s., nonsignificant; n.d., not detected). For all panels, results are shown as means with standard deviations from three independent experiments.

**FIG 4:**
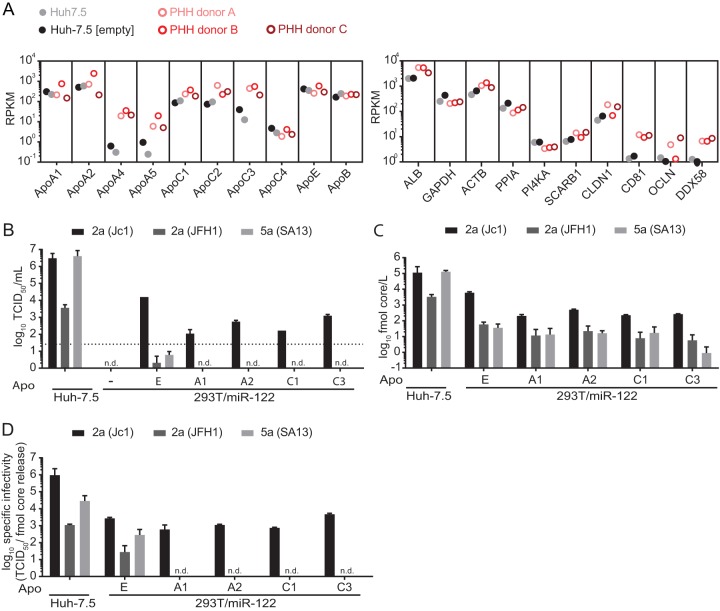
Preferential usage of ApoE over ApoA and ApoC during HCV assembly of different HCV constructs in 293T/miR-122 cells. (A) Relative mRNA expression levels are depicted as reads per kilobase per million reads (RPKM) of multiple exchangeable and nonexchangeable apolipoproteins in Huh-7.5 cells, lentivirus-transduced Huh-7.5 cells expressing an “empty” lentiviral vector (Huh-7.5 [empty]), and PHHs from three different donors. For comparison, RPKM values for the liver-specific host factor albumin (ALB), two housekeeping genes (glyceraldehyde-3-phosphate dehydrogenase [GAPDH] and β-actin [ACTB]), HCV replication factors cyclophilin A (PPIA) and phosphatidylinositol 4-kinase alpha (PI4KA), HCV entry factors scavenger receptor class B type 1 (SCARB1), claudin-1 (CLDN1), CD81, and occludin (OCLN), and the innate immune sensor RIG-I (DDX58) are presented on the right. (B) A subset of apolipoproteins that were highly expressed in Huh-7.5 cells/PHHs was selected to evaluate their ability to complement infectious virus production 48 h after HCV RNA transfection. Virus titers were assessed via limiting dilution assay, with the dotted line indicating the lower limit of quantification (LLOQ). (C) Release of core protein into the culture fluids was quantified by core-specific ELISA at 48 h posttransfection. (D) Specific infectivity was calculated based on core protein released into the supernatants and corresponding infectivity. For panels B, C, and D, means and standard deviations from two independent experiments are depicted (n.d., not detected).

The human ApoE gene has multiple allelic isoforms (ApoE2, -E3, and -E4), with the encoded proteins differing at one or two amino acid positions (residues 112 and/or 158) (48). These mutations have an impact on ApoE binding preferences toward different lipoprotein classes (49) and on low-density lipoprotein (LDL) receptor binding affinity (50), and these variants are associated with susceptibility to distinct disorders, including Alzheimer's disease (51) or type III hypolipoproteinemia (52). Hishiki et al. previously reported that HCV genotype 2a (JFH1) infectivity is influenced by association with distinct ApoE isoforms (53). In rescue experiments with ApoE-depleted Huh-7.5 cells, overexpression of ApoE2 resulted in poor recovery of infectious particle production, while ApoE3 and -E4 fully sustained infectivity. In contrast, using HCV *trans*-complemented particles (TCP) in mouse hepatoma cells, Long et al. demonstrated that all three human alleles of ApoE support HCV assembly with comparable efficiency (32). This discrepancy may be related to use of full-length versus *trans*-complemented particles. However, Long et al. used a Jc1 chimera whereas Hishiki et al. employed JFH1 wild-type viruses, suggesting that HCV usage of ApoE isoforms may also be strain specific. If so, viruses that do not effectively use ApoE3 may be attenuated in non-liver cells such as 293T cells ectopically expressing ApoE3. In Huh-7.5 cells, which, based on mapping of our transcriptome data (see Fig. 4A), express ApoE3, this may be less of an impediment for such isolates, as alternative exchangeable apolipoproteins (e.g., ApoA and ApoC variants) which are known to also support HCV assembly (40, 41) are coexpressed (see Fig. 4A) and thus may complement ineffective use of ApoE3. To address this, we generated stable 293T/miR-122 cell lines that ectopically express ApoE2, -E3, or -E4. Using an ApoE ELISA, we confirmed that all isoforms are expressed and secreted at similar levels (Fig. 3A). We did not observe significant differences in infectivity of the produced particles upon ectopic expression of other ApoE isoforms in 293T cells (Fig. 3B). Thus, at least in 293T cells, we were unable to detect strain-specific differences in use of allelic isoforms of ApoE. Moreover, ineffective use of ApoE3 does not explain why some strains, for instance, genotype 5a (SA13), do not efficiently produce infectious virus in these cells.

We next explored whether expression of additional host factors modulating lipid metabolism in human hepatocytes strain-specifically influences infectious virus production. The microsomal triglyceride transfer protein (MTTP) mediates triglyceride incorporation into nascent ER-luminal lipid droplets and is required for production and secretion of nascent ApoB-containing very-low-density lipoproteins (VLDLs) (54, 55). While it has been reported that inhibition of MTTP impedes HCV particle production, others suggested that HCV assembly is independent of MTTP (28, 56). To investigate this in the context of other HCV genotypes, we transfected 293T/miR-122/hApoE3/MTTP cells (33) with a selection of HCV chimeras that either yielded robust titers in 293T/miR-122/hApoE3 cells, such as genotypes 2b (J8) and 2a (Jc1), or failed to efficiently produce infectious particles, such as genotypes 5a (SA13) and 2a (JFH1). Protein expression of MTTP and the protein disulfide isomerase (PDI), a functional subunit of MTTP (57), was confirmed by immunoblotting (Fig. 3C). No significant differences were observed regarding infectious virus particle formation upon combined ApoE3/MTTP overexpression (Fig. 3D). Taken together, these data indicate that overexpression of MTTP in combination with ApoE3 does not modulate infectivity or rescue particle production of isolates that failed to form infectious particles when ApoE3 was expressed alone.

### ApoC or ApoA family members do not rescue virus production of HCV chimeras with poor assembly efficiency in 293T/miR-122/ApoE3 cells.

Primary human hepatocytes endogenously express numerous exchangeable apolipoproteins, and when endogenous ApoE is silenced in in Huh-7 cells, ectopic expression of various apolipoproteins boosts HCV assembly (40). Moreover, in non-liver-derived 293T/miR-122 cells, Jc1 HCV production is rescued by several exchangeable apolipoproteins (41), thus showing that HCV can use multiple apolipoproteins for virus production. Therefore, we speculated that some HCV strains may prefer alternative apolipoproteins over ApoE and because of this may be attenuated in 293T cells expressing ApoE3 compared to Huh-7.5 cells, where such alternative apolipoproteins are expressed. To address this, we first determined mRNA expression levels of multiple exchangeable apolipoproteins in primary human hepatocytes (PHHs) from three different donors and in two divergent batches of Huh-7.5 cells (i.e., parental Huh-7.5 cells and a population of Huh-7.5 cells transduced with a lentiviral vector) by transcriptome sequencing (RNA-seq). For control purposes, expression of housekeeping genes, hepatocyte markers, HCV entry and replication factors, and proteins of the innate immune sensing pathway is also presented. As depicted in Fig. 4A, global transcriptomic profiling revealed high mRNA expression levels of ApoE and ApoB in all samples. Furthermore, among the ApoE-related exchangeable apolipoproteins, ApoA1, -A2, -C1, -C2, and -C3 were also highly expressed in primary human hepatocytes and expressed at only slightly lower levels in the two Huh-7.5 cell batches. The abundance of mRNAs coding for ApoA4, -A5, and -C4 was clearly lower in PHHs and in the case of ApoA4 and -A5 was almost absent in the Huh-7.5 cell batches. To explore whether some HCV chimeras use ApoA or ApoC variants in preference to ApoE, we selected four variants highly expressed in both PHH and Huh-7.5 cells (ApoA1, -A2, -C1, and -C3) and ectopically expressed hemagglutinin (HA)-tagged variants in 293T/miR-122 cells (41). Comparable expression of these HA-tagged apolipoproteins was confirmed by an HA tag-specific ELISA (reference 41 and data not shown). As previously reported, ApoA1, -A2, -C1, and -C3 sustained infectious HCV particle production of Jc1 and compensated the function of ApoE during HCV assembly (Fig. 4B) (41). However, genotype 2a JFH1 and the genotype 5a (SA13) chimera that cannot utilize ApoE3 in 293T/miR-122 cells (Fig. 2B and 3C) were not rescued by these other exchangeable apolipoproteins (Fig. 4B). Since all these 293T cell lines expressed comparable levels of these apolipoproteins (41), these results confirm that genotype 2a (Jc1) preferentially uses ApoE, and they exclude a preference for these alternative apolipoproteins by other HCV strains. This conclusion was also supported by core protein measurements (Fig. 4C), since for each chimera, virus core release was greatest in ApoE-expressing 293T/miR-122 cells. Finally, for genotype 2a Jc1, we calculated the specific infectivity (Fig. 4D) and confirmed that specific infectivities of Jc1 particles produced in the presence of ApoA1, -A2, -C1, and -C3 are similar to that for ApoE-expressing 293T/miR-122 cells, albeit lower than that for Huh-7.5-derived particles.

### KO of endogenous ApoE expression in Huh-7.5 cells differentially affects virus production of HCV strains.

**FIG 5:**
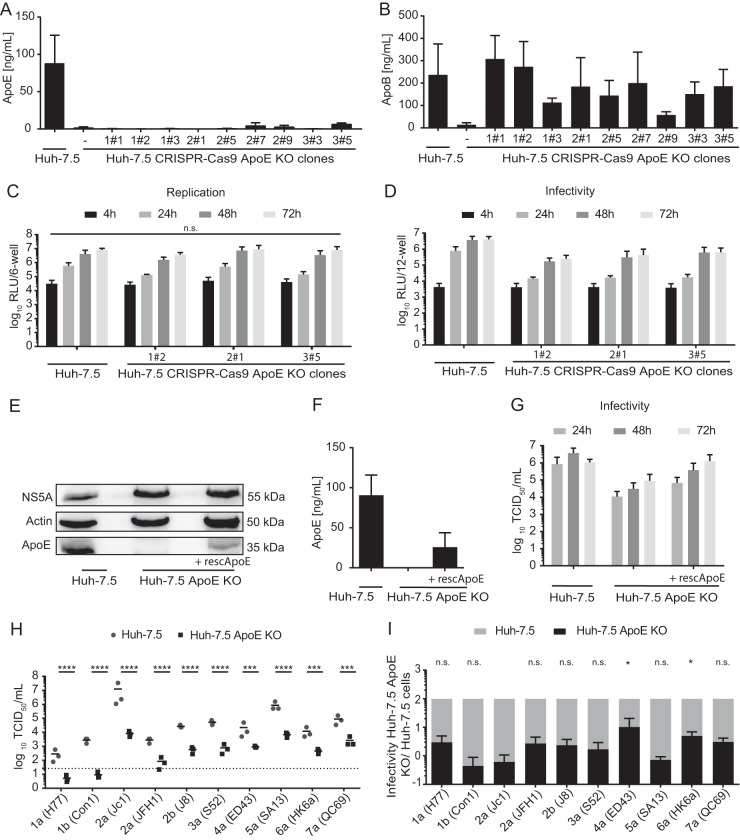
HCV replication and virus production in Huh-7.5 cell clones with ApoE knockout. (A and B) Individual Huh-7.5 clones deficient in endogenous ApoE expression were generated via CRISPR/Cas9-mediated knockout, subcloned, and characterized for ApoE (A) or ApoB secretion (B) into the culture fluids by commercially available ApoE and ApoB ELISAs. Means and standard deviations from at least two independent experiments are shown. (C) Three knockout cell clones were transfected with JcR2A HCV RNA encoding a Renilla luciferase reporter. At 4 h, 24 h, 48 h, and 72 h posttransfection, HCV RNA replication was monitored by luciferase measurements in the cell lysates. Luciferase activity is given in relative light units per well of a 6-well plate (RLU/6-well). Means and standard deviations from at least three independent experiments are depicted. Differences in RLU were compared among KO clones to Huh7.5 at each of the indicated time points (n.s., not significant). (D) Cell-free cell culture supernatants of the cells used for panel C were used to inoculate naive Huh-7.5 cells, and infectivity was determined by luciferase assay at 72 h postinoculation from three independent repetitions. Based on the data sets presented, cell clone 1#2 was selected for further analyses. (E to G) Endogenous ApoE KO was restored by ectopic expression of a C-terminally HA-tagged ApoE3 variant. Intracellular ApoE KO or overexpression of recombinant ApoE was confirmed by immunoblotting (E) and secretion of ApoE into the cell culture supernatant by ApoE-specific ELISA (F). Infectious virus production upon transfection with HCV Jc1 RNA was quantified by a limiting-dilution assay (G). Virus replication after transfection was assessed by immunoblotting of intracellular NS5A protein (E). For panel G, results from three independent experiments are shown. (H) The selected ApoE KO cell clone 1#2 and parental Huh-7.5 cells were transfected with RNA of JFH1 and nine chimeric HCV constructs, and at 48 h posttransfection, cell-free supernatants were used to inoculate naive Huh-7.5 cells. Infectivity was determined by limiting-dilution assay. The dotted line represents the lower limit of quantification (LLOQ) of the assay; independent repetitions are indicated as solid dots with a bar displaying the mean. Mean TCID_50_s in Huh-7.5 cells were compared to infectivity in the KO cell line for each strain (****, *P* < 0.0001; ***, *P* < 0.001 [by 2-way ANOVA followed by Sidak's multiple-comparison test]). (I) The efficiency of infectious virus particle release (E) from subcloned Huh-7.5 ApoE KO cells was compared to that from parental Huh-7.5 cells, which was set to 100%. The mean specific infectivity in Huh-7.5 cells was compared to that in ApoE KO cells for each strain (*, *P* < 0.05; n.s., not significant [by 2-way ANOVA followed by Sidak's multiple-comparison test]).

Next, we assessed ApoE usage by HCV strains in Huh-7.5 cells. To this end, we knocked out ApoE expression in these cells by clustered regularly interspaced short palindromic repeat (CRISPR)/Cas9 and examined virus production of JFH1 and nine HCV chimeras (Fig. 5). First, we characterized several Huh-7.5 ApoE knockout (KO) subclones regarding secretion of ApoE (Fig. 5A), secretion of ApoB (Fig. 5B), HCV RNA replication (Fig. 5C), and virus production (Fig. 5D) after transient transfection with a JcR2a Renilla luciferase-expressing reporter virus. Among all subclones characterized, we chose clone 1#2 for further analysis, because it displayed a knockout of ApoE expression but essentially normal ApoB secretion. Moreover, HCV RNA replication in this subclone was only marginally affected, while secretion of infectious progeny, as determined by transduction of luciferase activity, was reduced ca. 22-fold compared to parental Huh-7.5 cells (Fig. 5C and D). To confirm that the assembly defect of this subclone was caused by lack of ApoE, we rescued ApoE expression in this subclone by lentiviral gene transfer. Restoration of ApoE expression was confirmed by immunoblotting (Fig. 5E) and by an ApoE-specific ELISA (Fig. 5F). Upon transfection of parental Huh-7.5 cells or Huh-7.5 ApoE KO 1#2 cells with or without ApoE rescue with Jc1 we observed comparable accumulation of NS5A protein (Fig. 5E), suggesting similar transfection, translation, and replication efficiency in these cell lines. Importantly, restoration of ApoE expression in the Huh-7.5 ApoE KO 1#2 cells increased infectious virus production 5- to 10-fold between 24 and 27 h posttransfection (Fig. 5G). These data confirm previous results published by others and highlight the importance of ApoE during HCV assembly, while excluding other clonal effects of CRISPR/Cas9-mediated knockout cells.

Next we transfected the selected Huh-7.5 ApoE knockout clone with HCV RNA of JFH1 and all nine chimeras and compared virus production relative to that observed upon transfection of parental Huh-7.5 cells (Fig. 5H and I). All chimeras yielded significantly less infectious progeny upon transfection of the Huh-7.5 ApoE KO cell line than upon transfection of the parental Huh-7.5 cells. However, when we calculated the ratio of infectivity released from Huh-7.5 and Huh-7.5 ApoE KO cells for each of these chimeras, we observed significant differences. Infectious virus production by the genotype 2a (Jc1), genotype 1b (Con1), and genotype 5a (SA13) chimeras was impaired more than 100-fold in the Huh-7.5 ApoE KO cells. In contrast, 4a (ED43) and 6a (HK6a) chimeras were significantly less affected by knockout of ApoE than 2a (Jc1) and displayed only 10- and 20-fold-reduced virus production (Fig. 5I). Notably, these strain-dependent differences in ApoE usage between Huh-7.5 and Huh-7.5 ApoE KO cells did not directly mirror the differences in ApoE usage of these strains between 293T/miR-122/ApoE3 and Huh-7.5 cells. For instance, the ratio of 2a (Jc1) and 4a (ED43) virus production between Huh-7.5 and 293T/miR-122/ApoE3 cells was similar (Fig. 2E and F), while it was significantly different between Huh-7.5 and Huh-7.5 ApoE KO cells (Fig. 5H and I). In contrast, 2a (Jc1) and 5a (SA13) exhibited significantly different assembly efficiencies with Huh-7.5 and 293T/miR-122/ApoE3 cells (Fig. 2E and F), while both chimeras were affected to similar levels by knockout of ApoE in the Huh-7.5 background (Fig. 5H and I). Taken together, these results show that all HCV chimeras depend on ApoE for virus production. They also reveal that HCV chimeras are differentially susceptible to knockout of ApoE in Huh-7.5 cells and that they display differential efficiency of virus production in non-liver cells when only ApoE3 is expressed. Therefore, these results point toward differences in the fine-tuning of HCV assembly by host factors expressed in human liver and non-liver cells.

## DISCUSSION

Here we used JFH1 and nine different HCV chimeras representing all seven HCV genotypes to explore strain-dependent apolipoprotein usage during HCV assembly (43–47). These constructs have common viral nonstructural proteins NS3 to NS5B derived from the JFH1 strain. Thus, differences in virus production can be related to different functional properties in the structural proteins core, E1, and E2 as well as the p7 ion channel protein, as well as a portion of or the entire NS2 protein. On the one hand, we took advantage of 293T cells, a human kidney-derived cell line that is refractory to HCV RNA replication and virus production unless key liver-specific cofactors (miR-122 and ApoE) are provided in *trans* (33, 34). Moreover, unlike liver cells, these cells do not secrete human lipoproteins and lack essential components of lipoprotein production and secretion (e.g., MTTP and ApoB). Thus, these cells represent a minimal host environment for HCV assembly, where the role of individual host factors in assembly can be examined by complementation approaches. On the other hand, we used Huh-7.5 cells, a human hepatocellular carcinoma cell line that is highly permissive for HCV replication and virus production and that is typically used as the best available authentic background for HCV cell culture studies. Moreover, like primary human hepatocytes (Fig. 3), these cells express various exchangeable and nonexchangeable apolipoproteins, and they secrete lipoproteins decorated with ApoE and ApoB. Thus, this host cell background mimics conditions more closely related to primary human hepatocytes and also permits assessment of the contributions of various lipoproteins during HCV assembly. Using these models, we firmly established that all examined HCV chimeras produce HCV particles in a strictly ApoE-dependent manner, since none of the chimeras was able to produce infectious virus in 293T cells lacking ApoE expression (Fig. 2B). Moreover, knockout of ApoE expression in the highly permissive Huh-7.5 cell line significantly reduced infectious virus production by all chimeras (Fig. 5H). Unexpectedly, we observed strain-dependent differences regarding ApoE usage in these two models. First, parental genotype 2a (JFH1), 1a (H77), 1b (Con1), and 5a (SA13) chimeras are essentially unable to produce infectious HCV particles in 293T cells even if ApoE is ectopically expressed. In contrast, the genotype 2a (Jc1), 2b (J8), 4a (ED43), 6a (HK6a), and 7a (QC69) chimeras produce infectious HCV in this cellular background (Fig. 2B). By measuring intracellular core protein levels upon transfection of these chimeras, we excluded that these differences were due to divergent transfection, translation, and/or replication efficiencies. We also analyzed virus production of these chimeras in highly permissive Huh-7.5 cells and confirmed that the efficiency of assembly and release differs greatly between these chimeras (Fig. 2B) (43–47). While the low assembly efficiency of genotype 1a (H77) or 1b (Con1) chimeras may explain why infectious virus production in the less permissive 293T/miR-122/ApoE3 cells was not detected, this does not explain why other chimeras, including 3a (S52) with robust virus production in the Huh-7.5 cells and, even more strikingly, genotype 5a (SA13) with very efficient assembly in Huh-7.5 cells produced extremely low virus titers in the 293T cell background. These results indicate that chimeras differ in their ability to utilize ApoE3 as a cofactor for virus production, suggesting strain- and cell type-dependent differences in ApoE3 usage.

One possibility is that the structure/function of ApoE3 differs between the liver cell line Huh-7.5 and 293T cells and that only some strains are able to cooperate with the “version” of ApoE3 expressed in 293T cells. Alternatively, other exchangeable apolipoproteins and/or liver cell-specific assembly cofactors expressed in Huh-7.5 cells but lacking in 293T cells may allow efficient HCV assembly of chimeras with poor ApoE3 usage in the Huh-7.5 cells, whereas such chimeras are attenuated in 293T cells, as such putative compensating factors are lacking. Following these hypotheses, we attempted to rescue inefficient virus production of HCV chimeras in 293T cells by ectopic coexpression of MTTP together with ApoE3. Alternatively, we expressed different ApoE isoforms or other exchangeable apolipoproteins. The latter are highly expressed in Huh-7.5 cells and in primary human hepatocytes (Fig. 4A), and they are known to function in HCV assembly. Thus, they were candidates for compensating for poor usage of ApoE3 in Huh-7.5 cells. At least for genotype 2a (JFH1), it had been reported that this HCV strain differentially uses ApoE isoforms (53). Thus, it was possible that the chimeras poorly assembling in 293T cells expressing ApoE3 may be attenuated because of inefficient use of this specific ApoE allele. In Huh-7.5 cells that also express ApoE3, this impediment may be overcome due to coexpression of other exchangeable apolipoproteins that complement assembly.

Our experiments revealed that MTTP coexpression does not modify virus production of JFH1 and selected HCV chimeras in 293T cells (Fig. 3B). Moreover, they indicated that all tested virus constructs used ApoE isoforms to a similar degree. Finally, except for genotype 2a (Jc1), all tested HCV chimeras examined were unable to use ApoA1, -A2, -C1, or -C3 for assembly in the 293T background. Based on these observations, we can rule out that those chimeras with poor assembly efficiency in 293T/miR-122/ApoE3 cells (e.g., genotype 5a [SA13]) preferentially use alternative ApoE alleles or ApoA1, -A2, -C1, or -C3 instead of ApoE3. Therefore, other factors lacking in 293T cells and needed by these strains for efficient assembly may be required. Alternatively, these strains may require concomitant expression of several apolipoproteins together. The 293T system should be a useful tool to dissect these requirements in more detail.

The strain-dependent differences of ApoE3 usage by HCV in 293T cells described here do refer not only to assembly and release of virus particles but also to the properties of released virus particles. Most chimeras released viruses with comparable specific infectivity from 293T/miR-122/ApoE3 and Huh-7.5 cells; however, particularly genotype 5a (SA13) viruses released from 293T/miR-122/ApoE3 cells were much less infectious than those liberated from Huh-7.5 cells (Fig. 2D). It will be interesting to explore why these particles are less infectious. One possibility is that this chimera is not effectively loading ApoE onto nascent virus particles, which in turn would reduce the specific infectivity of released HCV given the important role of particles associated with ApoE for HCV attachment and cell entry (25, 27, 28).

Finally, we confirmed HCV strain-dependent ApoE usage in the context of Huh-7.5 hepatocellular carcinoma cells, which are typically used in HCV *in vitro* studies. In these cells also, strain-dependent usage of ApoE was observed. In the case of genotypes 2a (Jc1) and 5a (SA13), virus production was most heavily reduced (more than 100-fold), while chimeras 4a (ED43) and 6a (HK6a) were ca. 10-fold attenuated, which was significantly less than for Jc1.

Collectively, this study confirms that all tested HCV strains assemble infectious progeny in an ApoE-dependent manner. However, we demonstrate that there is a strain-dependent plasticity in ApoE usage, influenced in a cell type-dependent fashion. This differential ApoE usage affected not only assembly and release of infectious particles but also the properties of released virions. Thus, strain-dependent determinants of ApoE usage may have an impact on virus fitness at the level of assembly and cell entry. The use of the models described in this work in combination with other available full-length viruses apart from JFH1 to further dissect these differences should provide interesting insights into the host factors that govern HCV assembly and infectivity, with possible implications for the natural course of HCV infection.

## MATERIALS AND METHODS

### Constructs.

The Renilla luciferase-encoding reporter virus JcR2a and the different HCV chimeric isolates used in this study to assess the ApoE dependency for infectious virus particle production have been designed and constructed as described previously (43–47, 58–60). Briefly, these chimeras are composed of the GT2a-derived JFH1 5′ nontranslated region (5′NTR), the 3′NTR, and the JFH1 NS3- to NS5B-coding region fused with the core to NS2 genes of J8 (GT2b) (43), S52 (GT3a) (47), ED43 (GT4a) (45), SA13 (GT5a) (44), HK6a (GT6a) (43), or QC69 (GT7a) (43). In the case of H77 (GT1a), Con1 (GT1b), and J6 (GT2a), chimeric constructs were used where the junction between the isolates is located downstream of the first transmembrane domain of NS2, as this crossover position had been found to permit higher levels of infectious virus production for chimeras involving these strains (46). Note that the J6 (GT2a) chimera with this crossover site is usually designated Jc1 and is named such here for simplicity. Collectively, the structural proteins encoded by these chimeras, including core, E1, E2, and p7, thus represent all major HCV genotypes and therefore serve as a model to analyze strain-specific function and host factor interactions during HCV assembly. In some cases, chimeras carry adaptive mutations which optimize virus production of these chimeras in cell culture. These point mutations are highlighted with asterisks in Fig. 1C.

The plasmids encoding the different human ApoE isoforms (pWPI_ApoE2_BLR, pWPI_hApoE3_BLR, and pWPI_ApoE4_BLR) were constructed via PCR-based site-directed mutagenesis and verified by Sanger sequencing as described in one of our previous studies (61). Detailed cloning strategies are available upon request.

### Sequence acquisition and alignment.

Representative HCV E1E2 sequences were downloaded from GenBank, trimmed, translated, and aligned according to overlying encoded amino acids utilizing the Clustal W tool in MEGA5 (62). Translated amino acid conservation plots for E1E2 were calculated using a 10-amino-acid sliding window in CLC Genomics Workbench v10.

### Molecular phylogenetic analysis.

The evolutionary relationships of HCV E1E2 nucleotide sequences were calculated using the maximum-likelihood method implemented in MEGA5 (62) based on the data-specific model (63). The tree with the highest log likelihood (−96679.1416) is shown. Initial trees for the heuristic search were obtained automatically as follows. When the number of common sites was <100 or less than one-fourth of the total number of sites, the maximum-parsimony method was used; otherwise, the BIONJ method with MCL distance matrix was used. To assess the significance of clades, the bootstrap approach was employed, whereby 1,000 pseudoreplicate trees were generated using the neighbor-joining method. The presented tree was generated under a GTR+I+Γ model of substitution: the discrete gamma distribution was used to model evolutionary rate differences among sites (4 categories [+Γ, parameter = 0.5021]). The rate variation model allowed for some sites to be evolutionarily invariable ([+I], 23.9884% sites). The tree is drawn to scale, with branch lengths proportional to the number of substitutions per site. All positions containing gaps and missing data were eliminated from the analysis. The analysis incorporated nucleotide sequences from 495 E1E2 sequences and a total of 1,062 sites.

### PHHs.

Primary human hepatocytes (PHHs), obtained from the Department of General, Visceral, and Transplant Surgery at the Hanover Medical School, were cultured as described elsewhere (64). Liver tissue was processed from three different donors undergoing partial hepatectomy and was obtained upon written informed consent (approved by the ethic commission of Hanover Medical School/Ethik-Kommission der MHH, 252-2008). RNA from PHHs and human hepatoma cells (Huh-7.5 and Huh-7.5 [empty]) was extracted using a NucleoSpin RNA kit according to the manufacturer's instructions (Macherey-Nagel). RNA quality checking was performed using an Agilent Bioanalyzer, and RNA-seq was performed using the Illumina HiSeq 2000 platform. Transcriptomic profiling was performed with a CLC Genomics Workbench v9 (Qiagen Arhaus). Raw Fastq files were mapped against the hg19 human reference genome with annotated gene locations and transcript information. Gene expression was calculated for individual transcripts as reads per kilobase per million bases mapped (RPKM).

### Cell culture and cell lines.

Huh-7.5 cells, Huh-7.5 ApoE KO cells, and 293T/miR-122 derivatives were cultured in Dulbecco's modified Eagle's medium (DMEM) (Invitrogen) supplemented with nonessential amino acids (Invitrogen), 2 mM l-glutamine (Invitrogen), and 10% fetal calf serum (FCS) (PAA Laboratories GmbH) (DMEM complete). For selection of positive cell clones, 5 μg/ml blasticidin (InvivoGen) or 2 μg/ml puromycin (Sigma) was added.

Lentiviral gene transfer to ectopically overexpress the different human ApoE variants (ApoE2, -E3, and -E4) in 293T/miR-122 cells (33) was performed as described elsewhere (65). In short, plasmids pCMV-ΔR8.74 (66) and pcz-VSV-G (67), and derivatives of pWPI (encoding the gene of interest) were transfected at a 3:1:3 ratio into 293T/miR-122 cells. At 48 h posttransfection, lentiviruses were harvested and used to transduce target cells. Subsequent selection was performed by adding corresponding antibiotics.

The generation of 293T/miR-122 derivatives that ectopically express alternative apolipoproteins (ApoA1, -A2, -C1, and -C3) (41) or the additional liver-derived host factor MTTP (293T/miR-122/hApoE/MTTP) (33) was described previously.

### Generation of CRISPR/Cas9 knockout cell line.

Huh-7.5 cells that stably express the Cas9 enzyme were generated by lentiviral gene transfer as described previously (65), and positive cells were selected by addition of 5 μg/ml blasticidin (InvivoGen). Guide RNA sequences targeting ApoE were designed with the tool at http://www.genome-engineering.org/crispr/?page_id=41, and oligonucleotides were synthesized at IDT Technologies. Oligonucleotides were annealed, phosphorylated, and upon BsmBI digestion subsequently cloned into BsmBI-digested pLKO5d.sgRNA.EFS.PAC. Cloning was verified by Sanger sequencing. Together with the packaging plasmids pVSV-G and pcMV_ΔR8-74, the guide RNA-containing construct was subsequently transfected into 293T cells to produce lentiviruses that were used to transduce Huh-7.5 Cas9 expressing cells as described previously (65). Selection of positive cells was performed by addition of 2 μg/ml puromycin (Sigma). Single-cell clones were generated by seeding the cells at a density of 0.5 cell per 96-well plate and incubated until colonies started to grow. ApoE knockout was characterized by ApoE-specific ELISA (Mabtech, Nacka Strand, Sweden). The plasmid encoding the Cas9 enzyme (pLKO5d.EFS.SpCas9.P2A.BSD) and the plasmid harboring the individual guide RNA sequences (pLKO5d.sgRNA.EFS.PAC) were kindly provided by Dirk Heckl from Hanover Medical School and published previously (68). Detailed cloning strategies are available upon request.

### *In vitro* transcription and transfection of HCV RNA.

*In vitro* transcripts of HCV chimeric isolates were generated as described elsewhere (69). RNA integrity and concentration were checked by spectrophotometry and agarose gel electrophoresis, respectively. Subsequent transfection into target cells was performed as described previously (65). Briefly, trypsinized cells were washed with phosphate-buffered saline (PBS) and resuspended at 1.5 × 10^7^ cells/ml in Cytomix (70) containing 5 mM glutathione and 2 mM ATP prior to transfection with 1 μg of HCV RNA. Cells were immediately transferred into 10 ml of DMEM complete, and 4 ml of the cell suspension was seeded per 6-cm dish; alternatively, cells were transferred to 16 ml DMEM complete, and 3 ml of the suspension was seeded per well (6-well dish).

### Quantification of virus infectivity.

For the HCV chimeric isolates, extracellular viral titers were determined via endpoint dilution assay (TCID_50_) (https://www.klinikum.uni-heidelberg.de/fileadmin/inst_hygiene/molekulare_virologie/Downloads/TCID50_calculator_v2_17-01-20_MB.xlsx) (71). For infection assays using the Renilla luciferase reporter virus JcR2a, naive Huh-7.5 cells were seeded at a density of 8 × 10^4^ cells per well (12-well plate) at 24 h prior to infection with Renilla luciferase-conditioned cell culture supernatants. Luciferase expression was quantified at 72 h postinfection by cell lysis with addition of Milli Q water and a single freeze-thaw cycle, followed by addition of Coelenterazine (P. J. K. GmbH).

### ELISA.

To quantify secreted core protein, conditioned cell culture medium at 48 h posttransfection was inactivated by addition of Triton X-100 (Roth) at a final concentration of 1% (vol/vol), and core protein was quantified with a diagnostic kit (Architect Anti-HCV; Abbott). For quantification of intracellular core protein, cells were washed with PBS at 48 h posttransfection, scraped into 1.5-ml tubes, and centrifuged for 5 min at 1,000 × *g* at 4°C. Pellets were resuspended in DMEM complete and subjected to five cycles of freezing and thawing in liquid nitrogen followed by an additional centrifugation at 10,000 × *g* for 10 min at 4°C to remove cell debris. Upon addition of Triton X-100 (Roth), core amounts were also quantified with a diagnostic kit (Architect Anti-HCV; Abbott). Amounts of human ApoE and human ApoB100 were determined with commercially available ELISA kits according to the manufacturer's instructions (Mabtech, Nacka Strand, Sweden).

### Immunoblotting.

Intracellular expression of viral and cellular proteins was quantified as described previously (33) using anti-human PDI (1:1,000; Abcam) and anti-mouse–horseradish peroxidase (HRP) (1:20,000; Sigma) antibodies. Human MTTP was detected by probing with anti-human MTTP (1:1,000; Abcam) and anti-rabbit-HRP (1:15,000, Abcam) antibodies. For human ApoE, anti-ApoE (1:1,000; Calbiochem) and anti-goat–HRP (1:15,000; Abcam) antibodies were used. The viral protein NS5A was detected with anti-NS5A (9E10; 1:2,000) and anti-mouse–HRP (1:20,000; Sigma) antibodies. An anti-β actin antibody (1:2,000; Sigma), detected with an anti-mouse–HRP antibody (1:20000; Sigma), served as loading control.

### Statistics.

Data were analyzed using GraphPad Prism V.6.0b (GraphPad Software, La Jolla, CA, USA). Statistical analysis was performed by 1- or 2-way analysis of variance (ANOVA) followed by Sidak's multiple-comparison test or multiple *t* tests corrected with the Holm-Sidak method (****, *P* < 0.0001; ***, *P* < 0.001; **, *P* < 0.01; *, *P* < 0.05; n.s., nonsignificant [*P* > 0.05]; n.d., not detected).
